# A long-term survival case treated with conversion surgery following chemotherapy after diagnostic metastasectomy for pancreatic cancer with synchronous liver metastasis

**DOI:** 10.1186/s40792-017-0409-9

**Published:** 2017-12-29

**Authors:** Mitsuhiro Shimura, Masamichi Mizuma, Hiroki Hayashi, Akiko Mori, Tomoyoshi Tachibana, Tatsuo Hata, Masahiro Iseki, Tatsuyuki Takadate, Kyohei Ariake, Shimpei Maeda, Hideo Ohtsuka, Naoaki Sakata, Takanori Morikawa, Kei Nakagawa, Takeshi Naitoh, Takashi Kamei, Fuyuhiko Motoi, Michiaki Unno

**Affiliations:** Department of Surgery, Graduate School of Medicine, Tohoku University, 1-1 Seiryomachi, Aobaku, Sendai, 980-8574 Japan

**Keywords:** Pancreatic cancer, Liver metastasis, Conversion surgery, Chemotherapy, Diagnostic metastasectomy

## Abstract

**Background:**

Pancreatic cancer with distant metastases is classified as “unresectable,” for which the standard treatment is systemic chemotherapy. The effectiveness of radical resection for pancreatic cancer with distant metastases is unknown. Here, we report a case of long term survival treated with conversion surgery following chemotherapy after diagnostic metastasectomy for pancreatic cancer with synchronous liver metastasis.

**Case presentation:**

A 73-year-old man was referred to our hospital to examine and treat for cancer of the pancreatic body. Computed tomography (CT) scan revealed a 26-mm hypovascular tumor in contact with the common hepatic artery (CHA) (> 180°), the celiac artery (< 180°), and portal vein at the pancreatic body. Resectability was determined as “borderline resectable.” Two courses of gemcitabine plus S-1 combination therapy (GS) were administered as neoadjuvant chemotherapy (NAC). CT scan showed tumor shrinkage (21 mm), determined as stable disease (SD) according to Response Evaluation Criteria in Solid Tumors (RECIST). Although the abdomen was opened for radical resection, a small nodule on the liver was detected and removed. Since the nodule was diagnosed as adenocarcinoma by intraoperative frozen section, resection of the primary tumor was not performed. After three subsequent courses of GS therapy, no distant metastases were detected under radiological findings. Distal pancreatectomy with celiac artery resection (DP-CAR) was performed as radical surgery 6 months after the initial diagnosis. Histological diagnosis was well-differentiated tubular adenocarcinoma, showing ypT1 ypN1 M1 stage IV, negative surgical margin (R0), and grade III in the Evans classification. S-1 was administered every other day from 6 months after resection up to the present. The patient has been alive with no recurrence for 5 years after the initial diagnosis and 4.5 years after the resection.

**Conclusion:**

There is a case that received survival benefits from conversion surgery following chemotherapy after diagnostic metastasectomy in pancreatic cancer with synchronous liver metastasis.

## Background

Pancreatic cancer with distant metastases (M1) is classified as “unresectable,” for which the standard treatment is systemic chemotherapy. Although pancreatic resection and metastasectomy for M1 pancreatic cancer are suggested to be safe [1, 2], the prognostic contribution of radical surgery for M1 pancreatic cancer is unclear. Recently, reports on the effectiveness of conversion surgery after chemotherapy or chemoradiotherapy for initially unresectable locally advanced or M1 pancreatic cancer have been increasing due to the development of anticancer drugs [3, 4]. On the other hand, there are few reports of multidisciplinary treatment using a combination of radical resection and pre/post-operative chemotherapy for synchronous M1 pancreatic cancer. Here, we report a long-term survival case treated with a combination of conversion surgery following chemotherapy after diagnostic metastasectomy for pancreatic cancer with synchronous liver metastasis, showing 4.5-year recurrence-free survival and 5-year survival after initial diagnosis.

## Case presentation

**Fig. 1:**
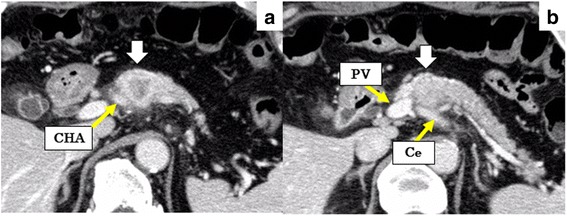
Abdominal CT scan at the initial diagnosis at the previous hospital. The white arrows indicate the pancreatic tumor. **a** A 26-mm hypovascular tumor in contact with the common hepatic artery (CHA) (> 180°) was seen in the pancreatic body. **b** The main tumor contacted the PV and bifurcation of Ce. CHA common hepatic artery, Ce celiac artery, SA splenic artery, PV portal vein

**Fig. 2:**
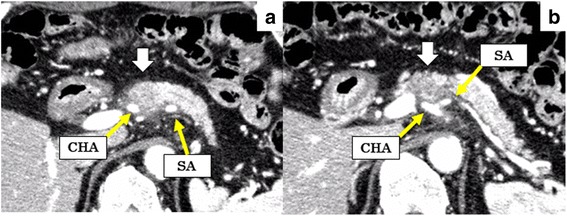
Abdominal CT scan after two courses of GS. The white arrows indicate the pancreatic tumor. **a** The size of the main tumor was reduced from 26 mm to 21 mm. The abnormal shadow of the soft tissue around CHA did not increase. **b** The abnormal shadow around the bifurcation of Ce did not change. CHA common hepatic artery, Ce celiac artery, SA splenic artery

**Fig. 3:**
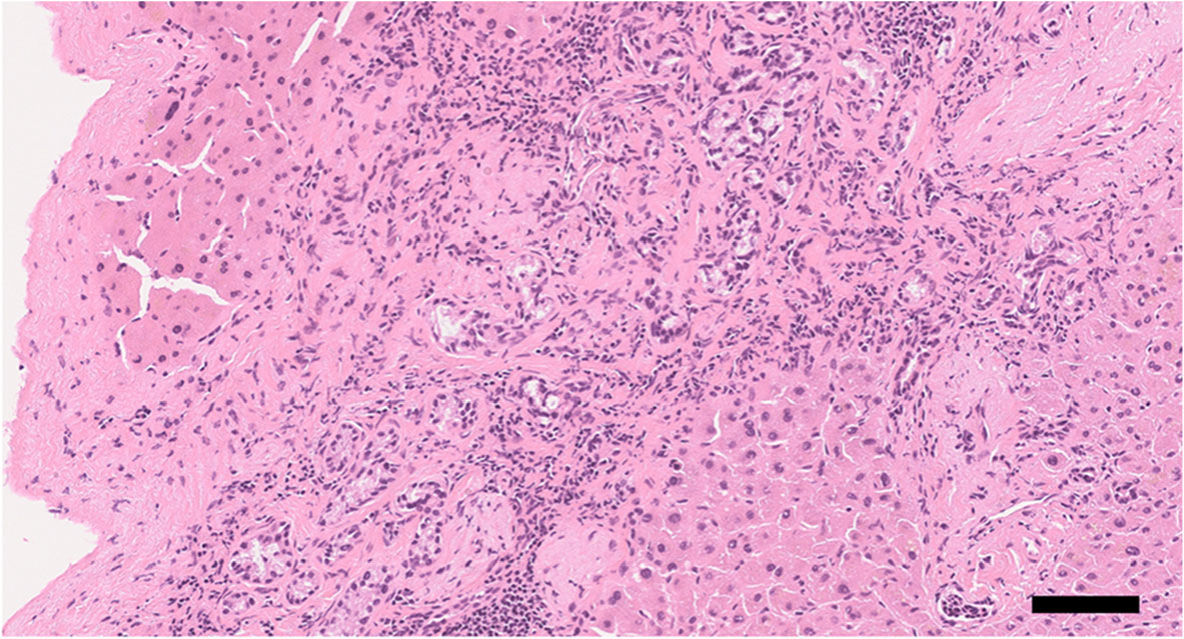
Intraoperative frozen sections. Adenocarcinoma cells invading the liver. Hematoxylin and eosin staining (×200, bar; 100 μm)

**Fig. 4:**
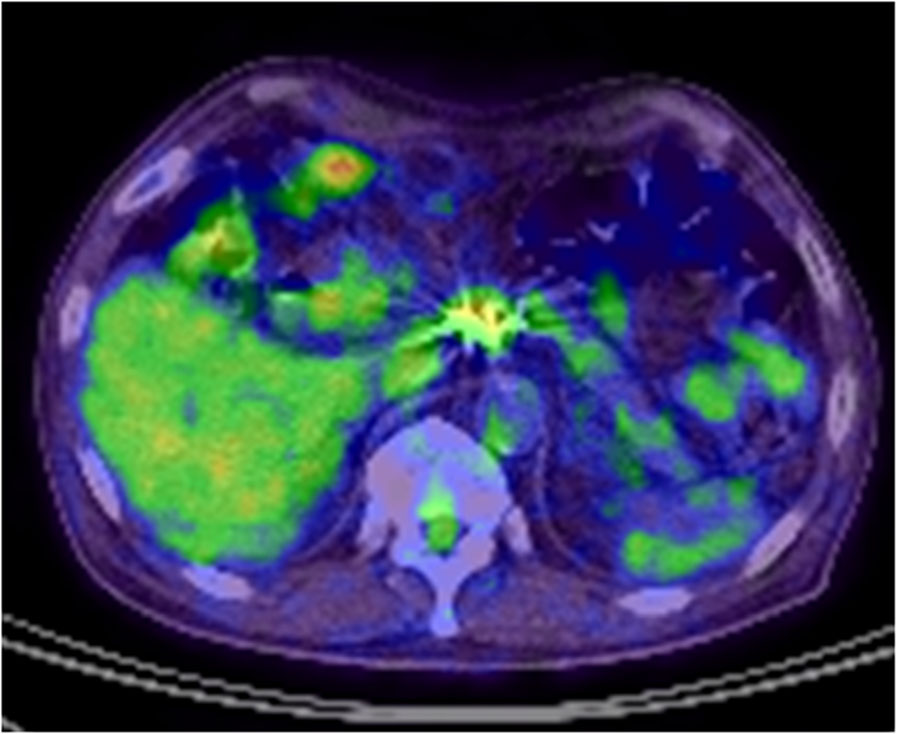
FDG-PET after three subsequent courses of GS. The main tumor of the pancreatic body had an abnormal accumulation of SUV max 3.8. Distant metastasis was not detected

**Fig. 5:**
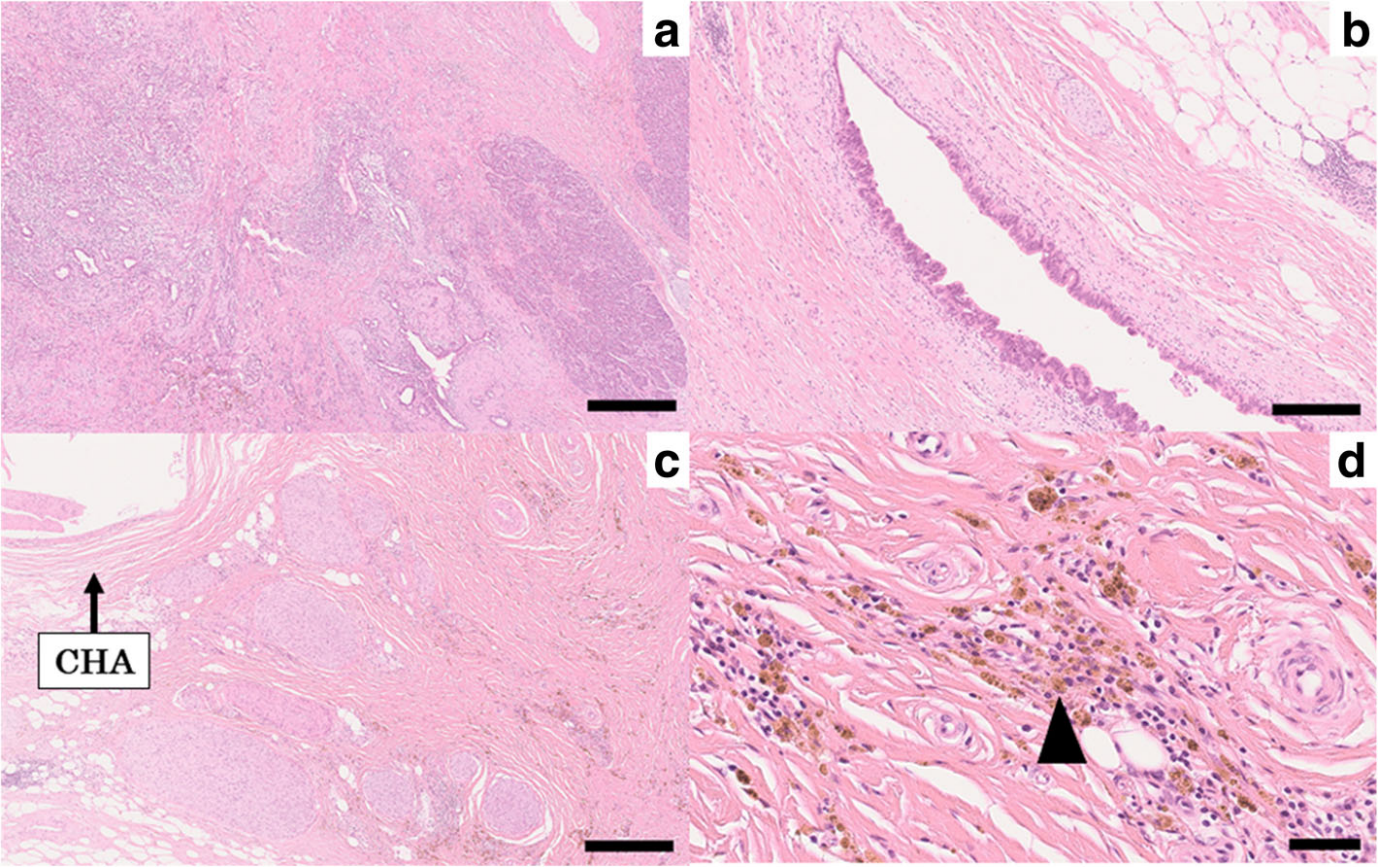
Representative histological photographs of resected specimen (hematoxylin and eosin staining). **a** The viable adenocarcinoma cells invaded into pancreatic tissue with the formation of various ductal structures (×50, bar; 500 μm). **b** The tumor cells extended to the main pancreatic ducts (×100, bar; 250 μm). **c** The tissue around the CHA was replaced with fibrous tissue (×50, bar; 500 μm). **d** The fibrous tissue included many histiocytes containing hemosiderin (arrowhead) (×400, bar; 50 μm)

A 73-year-old man visited a previous hospital due to upper abdominal pain. The patient had a past history of diabetes mellitus, hypertension, hyperlipidemia, and vestibular schwannoma. Abdominal computed tomography (CT) scan revealed wall thickening of the swollen gallbladder and a 26-mm hypovascular tumor in contact with the common hepatic artery (CHA) (> 180°), the celiac artery (< 180°), and portal vein at the pancreatic body (Fig. 1). The patient was diagnosed as pancreatic body cancer, and acute cholecystitis had also occurred. Laparoscopic cholecystectomy was performed for the acute cholecystitis, and simultaneous washing cytology was negative. After that, the patient was referred to our hospital for further examination and treatment of the pancreatic cancer. All of the examined tumor markers were within the normal range (CEA 1.7 ng/ml, CA19-9 2.0 ng/ml, DUPAN-2 60 U/ml, SPan-1 1.0 U/ml, CA-125 12.4 U/ml). Since serum CA19-9 was at an undetectable level, Lewis antigen of the patient might be negative. No liver metastasis was found by gadoxetic acid-enhanced magnetic resonance imaging (EOB-MRI). According to the National Comprehensive Cancer Network (NCCN) Guidelines Version 2.2016, the resectability was determined to be “borderline resectable.” Distal pancreatectomy with celiac artery resection (DP-CAR) after neoadjuvant chemotherapy (NAC) was planned. Two courses of gemcitabine (GEM) plus S-1 combination therapy (GS) were performed as NAC [5]. GEM was given at a dose of 1000 mg/m^2^ on days 1 and 8 of a 21-day cycle, and S-1 was administered at a dose of 40 mg/m^2^ twice daily on days 1–14 followed by a 7-day rest. Enhanced CT scan after NAC showed a 21-mm pancreatic tumor, indicating stable disease (SD) in the Response Evaluation Criteria in Solid Tumors (RECIST) (Fig. 2). EOB-MRI and fluorine-18 fluorodeoxyglucose (FDG) positron emission tomography (PET) after NAC revealed no distant metastases. Coil embolization of the CHA and left gastric artery was done 3 days before surgery in order to prevent ischemic changes of the liver and stomach. Laparotomy was performed for DP-CAR 3 months after the initial diagnosis. A 3-mm tumor was detected on the surface of the liver (segment IV) and was removed. The liver tumor was diagnosed as adenocarcinoma by intraoperative frozen sections (Fig. 3). Therefore, resection of the primary tumor was not performed. Three subsequent courses of GS were administered. Afterward, no distant metastases were seen in CT scan, MRI, or FDG-PET (Fig. 4). The RECIST was unevaluable due to the artifact of the coil. DP-CAR was performed 6 months after the initial diagnosis. Intraoperative findings showed negative washing cytology and no distant metastases. The operative time was 429 min, and the amount of bleeding was 1150 ml. The proliferation of atypical cells with low papillary structure and disruption of the cell polarity was observed histologically in the main pancreatic duct. The tumor cells had nuclear enlargement and nuclei of irregular shape in the invasive portion, showing degeneration by chemotherapy. The histological diagnosis was well-differentiated tubular adenocarcinoma (Fig. 5). TNM classification of the Union for International Cancer Control (UICC) (7th edition) was ypT1 ypN1 M1, Stage IV. No carcinoma cells were detected in the surgical margin (R0). Because of the widespread fibrous tissue and some residual tumor cells, the histopathological effect of chemotherapy was determined as grade III in the Evans classification [6]. Although grade C of delayed gastric emptying (DGE), defined by the International Study Group of Pancreatic Surgery (ISGPS), developed after the surgery, the patient was discharged after 91 postoperative days. S-1 was administered every other day at a dose of 100 mg/day from 6 months after the resection up to the present. The patient has been alive with no recurrence for 5 years from an initial diagnosis and 4.5 years after DP-CAR.

### Discussion

Pancreatic cancer is one of the most lethal malignancies. Gillen et al., based on an analysis of 111 studies from 1966 to 2009, reported that the proportions of resectable pancreatic cancer, locally advanced/unresectable pancreatic cancer, and metastatic pancreatic cancer at the initial diagnosis were approximately 10–20, 30–40, and 50–60%, with a median survival time (MST) for metastatic pancreatic cancer of only 5–9 months [7]. Recently, two powerful regimens, oxaliplatin, leucovorin, irinotecan, plus 5-fluorouracil (FOLFIRINOX) and GEM plus nab-paclitaxel (G+nab-P), are being used as the standard treatment for metastatic pancreatic cancer. MST has been reported to be 10.7–11.1 months with FOLFIRINOX [8, 9] and 8.5–13.5 months with G+nab-P [10, 11]. Although the development of chemotherapy has improved the prognosis of pancreatic cancer, long-term survival cases treated with chemotherapy alone are still rare. There are several reports about the effectiveness of resection for liver metastases in pancreatic cancer. It has been reported that liver resection for synchronous liver metastasis in pancreatic cancer did not contribute to improving the prognosis [12, 13]. On the other hand, Tachezy et al. has reported that synchronous resection of the primary tumor and liver metastases in pancreatic cancer prolonged survival compared with non-resection [14]. Although this study had a patient selection bias of a surgical indication for liver metastases, cases that received a survival benefit from surgical resection for primary tumor and liver metastasis exist, as in our case. Hackert et al. reported that pancreatic resection and metastasectomy for oligometastatic pancreatic cancers contributed to a prolonged survival of 10% in selected patients [1]. In this study, 4.7% of patients with liver metastasis underwent prior liver resection, followed by chemotherapy and pancreatic resection. Also, Buc et al. has described that a case of pancreatic cancer with a synchronous single liver metastasis has undergone conversion surgery following chemotherapy after diagnostic metastasectomy as in our case and has been alive without recurrence over 2 years from initial diagnosis [15]. Pancreatic cancer with small superficial liver oligometastases which are diagnosed at the time of surgery without detection by pre-operative imaging might be a good candidate for this strategy, namely conversion surgery after diagnostic metastasectomy.

There are few reports of conversion surgery with simultaneous metastasectomy after chemotherapy for pancreatic cancer with synchronous liver metastases. As far as we could determine, there were only two cases that underwent conversion surgery after FOLFIRINOX, reported by Schneitler et al. [16]. One has been alive for 24 months after the diagnosis and 18 months after the resection. The other has been alive for 17 months since the diagnosis and 9 months after resection with no recurrence. In these cases, conversion surgery with simultaneous metastasectomy might have contributed to the prolongation of survival. Further observation is needed to evaluate the long-term prognosis.

FOLFIRINOX or G+nab-P improves the prognosis of metastatic pancreatic cancer. Currently, the standard regimen for metastatic pancreatic cancer is FOLFIRINOX or G+nab-P. GS therapy showed a better response rate for patients with locally advanced or metastatic pancreatic cancer than GEM or S-1 monotherapy in the GEST study [17]. GS as neoadjuvant for resectable and borderline resectable pancreatic cancer could contribute to R0 resection and overall survival [5]. Currently, a phase III randomized controlled trial of neoadjuvant GS for resectable pancreatic cancer (Prep-02/JSAP-05; UMIN000009634) is ongoing.

FOLFIRINOX or G+nab-P is expected to increase the number of patients with metastatic pancreatic cancer well-controlled for a certain period and patients undergoing conversion surgery as in our case. The median progression-free survival (PFS) of FOLFILINOX and G+nab-P has been reported to be 5.6–6.4 months [5, 6] and 5.5–6.5 months [7, 8], respectively. It might be better to consider the PFS in the timing of conversion surgery.

## Conclusions

The present case received survival benefits from conversion surgery following chemotherapy after diagnostic metastasectomy for pancreatic cancer with synchronous liver metastasis.

## Abbreviations

CHA: Common hepatic artery
CT: Computed tomography
DGE: Delayed gastric emptying
DP-CAR: Distal pancreatectomy with celiac artery resection
EOB-MRI: Gadoxetic acid-enhanced magnetic resonance imaging
FDG: Fluorine-18 fluorodeoxyglucose
FOLFIRINOX: Oxaliplatin, leucovorin, irinotecan, plus 5-fluorouracil
G+nab-P: Gemcitabine plus nab-paclitaxel
GEM: Gemcitabine
GS: Gemcitabine plus S-1 combination therapy
ISGPS: International Study Group of Pancreatic Surgery
MST: Median survival time
NAC: Neoadjuvant chemotherapy
NCCN: National Comprehensive Cancer Network
PET: Positron emission tomography
PFS: Progression-free survival
RECIST: Response Evaluation Criteria in Solid Tumors
UICC: Union for International Cancer Control
